# Comment on: CG258 *Klebsiella pneumoniae* isolates without β-lactam resistance at the onset of the carbapenem-resistant Enterobacteriaceae epidemic in New York City

**DOI:** 10.1093/jac/dky472

**Published:** 2018-11-30

**Authors:** Kelly L Wyres, Margaret M C Lam, Kathryn E Holt

**Affiliations:** 1 Department of Biochemistry and Molecular Biology, Bio21 Molecular Science and Biotechnology Institute, University of Melbourne, Parkville, VIC, Australia; 2 London School of Hygiene and Tropical Medicine, London, UK

Sir,

We read with interest the recent article by Eilertson *et al*.,^1^ ‘CG258 *Klebsiella pneumoniae* isolates without β-lactam resistance at the onset of the carbapenem-resistant Enterobacteriaceae epidemic in New York City’, wherein the authors conduct an investigation of *K. pneumoniae* causing bloodstream infection in New York City in 1999, 2003–04, 2006, 2009 and 2013. However, we felt that readers would benefit from further discussion of the results in the context of what is known about clonal group (CG) 258, in particular from recent genomic analyses.

In the article by Eilertson *et al*.,^1^ CG258 was defined in the discussion section as ‘ST258 and its single allele variants’, referring to alleles of the seven-locus MLST scheme.^2^ Notably, this definition includes ST11, which: (i) has been shown to be the ancestor of ST258; (ii) differs from ST258 by a single allele (*tonB*); and (iii) is explicitly included in the definition of CG258 in numerous other studies.^2–5^

Genomic analyses by six independent groups have concluded that ST258 evolved from an ST11 ancestor^3–8^ into which an ∼1 Mbp sequence was imported from an ST442 strain.^3^^,^^6^ The imported region includes the capsule locus harbouring *wzi154*^3^^,^^6–8^ (referred to as *cps-2* in Eilertson *et al*.,^1^ now designated KL107 under the standardized nomenclature^9^) and the *tonB-79* allele, which replaced the *tonB-4* allele of ST11, converting it into ST258.^3^ Notably, in the article by Eilertson *et al*.^1^ the PCR used to screen for CG258 isolates targets the *tonB-79* allele of ST258, which is not present in ST11. Hence, the screen detects only the *tonB-79* subgroup of CG258 and is specifically unable to detect ST11 isolates, which may be the most informative members of CG258 in terms of their potential to reveal details of the early emergence of ST258.^3–8^

**Figure 1:**
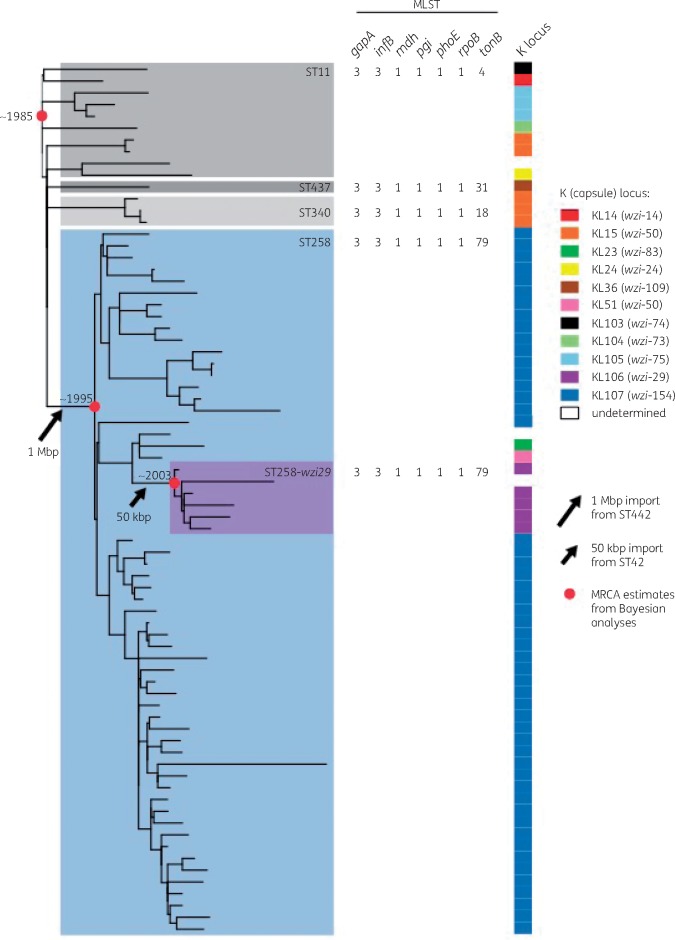
Evolutionary history of CG258. Phylogeny and K (capsule) locus data are reproduced from Lam *et al*.^10^ (outgroup-rooted and recombination-filtered maximum likelihood phylogeny inferred from core genome nucleotide variants). Clades corresponding to each chromosomal ST plus the ST258-*wzi29* subclade are highlighted and labelled, followed by the seven-gene MLST designation. K locus and *wzi* allele numbers are shown by coloured blocks according to the key. Red circles mark the hypothetical most recent common ancestors for which dates have been estimated in published Bayesian analyses: (i) the whole CG;^5^ (ii) all ST258;^4^^,^^5^ and (iii) ST258-*wzi29* subclade.^4^

Eilertson *et al*.^1^ report that the earliest ST258 isolates they identified (2003) carried *wzi154*, while some later isolates carried *wzi29* and occasionally other *wzi* alleles. This is as expected given the previously reported genomic data, which show that following the initial formation of ST258-*wzi154* (KL107), a subsequent ∼50 kbp recombination event occurred with an ST42 *K. pneumoniae*,^3^^,^^4^^,^^6–8^ importing a new capsule locus harbouring *wzi29* to form the *ST258-wzi29* subclade (referred to as *cps-1* in Eilertson *et al*.,^1^ designated KL106 under the standardized nomenclature^9^). The genomic comparisons therefore support a line of descent from ST11 to ST258-*wzi154* to ST258-*wzi29* (summarized in Figure 1). Molecular dating analyses estimate that ST258 emerged from ST11 in the mid-1990s^4^^,^^5^ and the ST258-*wzi29* subclade emerged ∼7–8 years later in the early 2000s.^4^

The detection of ST258-*wzi154* (KL107) in 2003, with ST258-*wzi29* (KL106) detected later, is therefore consistent with the prior data on the stepwise evolution of ST258. However, contrary to the statements in the manuscript of Eilertson *et al*.,^1^ these data do not suggest that KL107 (*wzi154*) was the initial capsule type of CG258, either in New York City or globally, as the study captured only ST258 and its direct derivatives that form just one subgroup of CG258 (as discussed above). The ancestral capsule type of the entire CG, or of the ST11 progenitor strain from which ST258 emerged through recombination, remains unknown because CG258 harbours extensive capsule locus diversity (e.g. see Figure 1).^3^^,^^4^^,^^9^^,^^10^
